# Serum S100B correlates with health-related quality of life and functional outcome in patients at 1 year after aneurysmal subarachnoid haemorrhage

**DOI:** 10.1007/s00701-022-05272-0

**Published:** 2022-06-24

**Authors:** Helena Aineskog, Conny Johansson, Robert Nilsson, Lars-Owe D. Koskinen, Peter Lindvall

**Affiliations:** Department of Clinical Sciences – Neurosciences, Umeå University, 901 87 Umeå, Sweden

**Keywords:** Subarachnoid haemorrhage, Modified ranking scale, EQ-5D-3L: EuroQoL health-related quality of life, S100B

## Abstract

**Background:**

Early, objective prognostication after aneurysmal subarachnoid haemorrhage (aSAH) is difficult. A biochemical marker would be desirable. Correlation has been found between levels of the protein S100 beta (S100B) and outcome after aSAH. Timing and clinical usefulness are under investigation.

**Methods:**

Eighty-nine patients admitted within 48 h of aSAH were included. Modified ranking scale (mRS), EuroQoL health-related quality of life measure (EQ-5D_index_) and EuroQoL visual analogue scale (EQ-VAS) values were evaluated after 1 year. S100B was measured in blood samples collected at admission and up to day 10.

**Results:**

S100B correlated significantly with EQ-5D_index_ and mRS, but not EQ-VAS at 1 year after aSAH. A receiver operating characteristic analysis for peak S100B values (area under the curve 0.898, 95% confidence interval 0.828–0.968, *p* < 0.0001), with a cutoff of 0.4 μg/l, yielded 95.3% specificity and 68% sensitivity for predicting unfavourable outcome. Dichotomized S100B (> 0.4 μg/l vs ≤ 0.4 μg/l), age and Hunt and Hess grading scale score (HH) were associated with unfavourable mRS outcome in univariate logistic regression analysis. Dichotomized S100B was the only variable independently correlated with unfavourable mRS outcome in a multivariate logistic regression analysis.

**Conclusions:**

For the first time, S100B was shown to correlate with mRS and health-related quality of life at 1 year after aSAH. Peak S100B can be used as a prognostic factor for unfavourable outcome measured as dichotomized mRS after aSAH. A peak value cutoff of 0.4 μg/l is suggested. Ethical approval no: 2013/366-31, 4th of February 2014.

## Introduction

Aneurysmal subarachnoid haemorrhage (aSAH) remains a major cause of morbidity and mortality. Although accounting for a small proportion of all strokes (5%), the early age of onset (40–60 years) and the poor prognosis result in loss of many productive life years [9]. The most common cause of spontaneous SAH is aneurysm rupture, with an incidence of 9 cases per 100,000 patients/year [9].

Factors associated with unfavourable outcome include high age, worse clinical condition on admission and amount of blood seen on first computed tomography (CT) scan [6, 12, 19]. The Hunt and Hess grading scale score (HH) can be used for grading the clinical condition on admission and is strongly correlated with poor outcome, but the instrument is based on clinical assessment, making it subject to interobserver variability and difficult to use in sedated patients. The Fisher grade can be used for rating the amount of blood seen on first CT scan after aSAH [3]. A biochemical marker to objectively and accurately assess and predict the clinical severity of SAH would be of great clinical value.

S100 beta (S100B), a calcium-binding protein of 9–14 kDa with a relatively short biological half-life, occurring primarily as homodimers, has been recognised as a reliable marker for brain tissue injury [22]. The protein is mainly concentrated in astrocytes and other glial cells within the central nervous system, but is also found in, e.g. adipocyte tissue and melanocytes. Elimination of S100B occurs only in the kidneys [22]. Stored S100B is released extracellularly from astrocytes affected by trauma or metabolic distress [4]. The majority of S100B in bodily fluids seems to come from dead or dying cerebral tissue [22].

S100B has been studied in relation to several conditions, such as traumatic brain injury, ischemic stroke and spontaneous SAH, with its concentration in serum found to correlate well with both outcome and extent of injury to the brain parenchyma [10, 15, 22].

There are data supporting the use of serum S100B for prognostication of functional outcome after aSAH [1, 2, 17]. Questions remain regarding the best timepoint to measure its levels, and if an average value or a peak value is more reliable [20]. No convincing correlation has been found between deterioration in vasospasm after SAH and higher levels of S100B [8].

To our knowledge, no previous attempts have been made to investigate the use of S100B to prognosticate long-term outcome measured as health-related quality of life.

We hypothesised that there might be an association between brain tissue injury measured as S100B after aSAH and health-related quality of life as well as functional outcome.

Our primary aim was to investigate if S100B could be used for prognostication of health-related quality of life and functional outcome 1 year after aSAH.

## Materials and methods

### Study population

Patients, aged ≥ 18 years, admitted to our unit between November 2014 and March 2018 within 48 h of ictus of aSAH from an aneurysm not previously treated were considered for inclusion. This retrospective study of prospectively collected data was planned a priori as part of a larger multi-centre study run in parallel, encompassing all neurosurgical centres in Sweden. A prospectively enrolled cohort of 103 consecutive patients was included in the study. Informed consent was obtained from all included patients or an appropriate next of kin (90 patients).

### Clinical and CT evaluation

At admission, clinical severity was evaluated using HH, as described elsewhere [5]. The aSAH diagnosis was confirmed through CT and CT angiography of the head or digital subtraction angiography. All patients eligible for treatment underwent endovascular intervention or surgery, as assigned by a board of neurosurgeons and neuroradiologists. The initial CT was reviewed by an independent neuroradiologist on site, blinded to S100B. The blood distribution was evaluated by a specially trained clinician (HA), blinded to all other data, using the Fisher grade [3]. The Fisher grade was dichotomized (dFG) by amount of blood into ‘FG0–3’, in case of a score 0–3 and ‘FG4’, in case of the score 4.

Follow-up was performed 1 year after aSAH. Functional outcome was assessed using the modified ranking score (mRS), determined based on a structured interview with the patient or next of kin performed by a trained research nurse [7, 24]. A dichotomization of mRS (dmRS) was used to define outcome as favourable (0–3) or unfavourable (4–6).

In this study, health-related quality of life was assessed using both EQ-5D_index_ and the visual analogue scale EQ-VAS. EQ-5D_index_ is a self-assessed, five-dimensional scoring system focusing on mobility, self-care, usual activities, pain/discomfort and anxiety/depression. Each dimension is scored at one of three levels (no, moderate or severe problems). The UK value set was used to calculate an EQ-5D_index_ value for each patient. This generates 243 possible health states, each represented by a single index value, EQ-5D_index_, where 0 represents death and 1 represents full health. EQ-VAS is a self-assessment instrument, ranging from 0 (worst imaginable health) to 100 (best imaginable health), and can only be used if the patient is alive. Next of kin would help with evaluating EQ-5D_index_ when possible, but not EQ-VAS.

### Chemicals and equipment

Venous blood samples were collected at admission to the neuro-intensive care unit, and every third day by a research nurse (during weekdays, 8 am to 4 pm) up to day 10. Patients admitted after S100B were introduced as a standard measure in the clinic (*n* = 58) and had samples taken daily in accordance with the clinical sampling protocol. Day 0 was defined as the first 24 h after the suspected timepoint of haemorrhage. If two samples were collected from a patient during the same time interval, e.g. 0–12 h, 0–24 h, the highest recorded value was included. Serum samples were analysed using a sandwich-biotinylated monoclonal S100B and second-stage ruthenium complex-based assay (ECLIA, Elecsys S100, Cobas 801, Roche Diagnostics Scandinavia). The method is established as a routine assay at the accredited laboratory at Umea University Hospital. According to the manufacturer, normal serum levels are ≤ 0.1 μg/l.

### Statistical analyses

IBM SPSS Statistics software (v 26) was used for analyses. Non-parametric tests were used as the data were not normally distributed. Mann-Whitney *U*-tests were used to compare demographics as well as S100B and Fisher grade. A Kruskal-Wallis test was performed to compare S100B levels between outcome groups. Spearman’s correlation was used to analyse correlations between S100B levels and outcome based on mRS, EQ-5D_index_ and EQ-VAS.

The optimal cutoff value of serum S100B levels for predicting unfavourable outcome and the corresponding sensitivity and specificity was determined based on a receiver operating characteristic (ROC) curve analysis and the corresponding area under the curve (AUC). A significance level of *p* ≤ 0.05 was used. Multivariate logistic regression was performed to assess the predictive capacity of S100B, as well as the other variables included. Clinically relevant, known predictors of functional outcome were chosen before performing the analysis. We only used predictors that were easily available or readily measured at admission to the neurosurgical department. *p* for the parameters included in the multivariate analysis was set at 0.05. An Omnibus test was run to reject the null hypothesis for the multivariate regression analysis and chi-squared and Hosmer and Lemeshow tests were used to identify a good model fit. Based on the ROC curve analysis, a dichotomized S100B variate was included, with S100B > 0.4 μg/l assumed to correlate with unfavourable outcome and S100B ≤ 0.4 μg/l assumed to correlate with favourable outcome.

Univariate logistic regression was performed for all considered variables, including those not used for the multivariate analysis. Univariate logistic regression was used to assess the predictive capacity of S100B levels, as well as other prognostic factors, for unfavourable clinical outcome and *p* was set to 0.01 for the analysis. Nagelkerke’s pseudo *R*^2^ was used to further explore the predictive capacity of S100B at different timepoints. A scatterplot was created to illustrate individual representation of EQ-5D_index_ in relation to peak S100B. We did not correct for multi-comparisons, as the most important results had a *p* < 0.01.

## Results

Amongst a total of 103 patients preliminarily included, 13 were excluded because no S100B samples were available. One patient withdrew from participation at follow-up. Twelve patients died before follow-up (four within a week, nine within a month, eleven within 3 months and all within 7 months). All 89 patients were included in the mRS evaluation. Eleven patients did not attend an EQ-5D_index_ evaluation and 23 could not perform the EQ-VAS. Follow-up was performed 1 year after aSAH, median 1.4 years in the favourable outcome group (min 0.9, max 3.4 years) and median 1.5 years in the unfavourable outcome group (min 0.9, max 3.3 years), without any significant difference between the two groups (*p* = 0.791). Clinical characteristics and differences in parameters between functional outcome groups are presented in Table 1. High age, multiple aneurysms and endovascular treatment correlated with unfavourable mRS outcome. The unfavourable outcome group had higher Fisher grades and HH. HH at admission was missing for 11 patients, who were admitted already sedated and intubated at arrival. Surgery was performed in 49 patients (55%) and 36 (40.5%) were treated with an endovascular approach. Four patients had no intervention due to poor clinical status. Favourable outcome, measured as dmRS 1 year after ictus, was seen in 72% (*n* = 64) of the patients.

**Table 1:** Clinical characteristics and differences between dichotomized mRS outcome groups (favourable and unfavourable). *Ant* anterior cerebral circulation, *Post* posterior cerebral circulation, *Endo* endovascular intervention, *Surg* surgery

		Total	Favourable	Unfavourable	*p*
Age (years)	Median (min–max)	60 (28–82)	58 (28–79)	65 (28–82)	0.006
Gender (male:female)	n:n	25:64	19:45	6:19	0.794
BMI (kg/m^2^)	Mean (min–max)	27 (17–49)	28 (17–49)	27 (20–43)	0.334
Hypertension (yes:no)	n:n	40:49	27:37	13:12	0.480
Ongoing smoking (yes:no)	n:n	37:49	24:40	13:9	0.087
Multiple aneurysms (yes:no)	n:n	20:69	10:54	10:15	0.022
Aneurysm location (Ant:Post)	n:n	74:15	55:9	19:6	0.345
Treatment modality (Endo:Surg)	n:n	36:49	23:41	13:8	0.045
Fisher grade (I:II:III:IV)	n:n:n:n	0:1:22:66	0:1:22:41	0:0:0:25	-
HH grade (I:II:III:IV:V)	n:n:n:n:n	9:28:17:17:7	8:28:15:8:0	1:0:2:9:7	-
mRS (0:1:2:3:4:5:6)	n:n:n:n:n:n		33:15:7:9:0:0:0	0:0:0:0:10:3:12	

### Outcome

EQ-5D outcome at 1-year follow-up is shown in Table 2. The mean EQ-5D_index_ was 0.58 ± 0.38 (*n* = 78) and mean EQ-VAS was 72.1 ± 21.8 (*n* = 66). Worse outcome for both EQ-5D_index_ and mRS correlated with higher serum levels of S100B (Spearman’s correlation), as shown in Table 3. No significant correlation was found for EQ-VAS. Strong associations were seen between EQ-5D_index_ and mRS, respectively, and dichotomized S100B peak levels (> 0.4 μg/l vs ≤ 0.4 μg/l) with individual sampling in the first 0–12 h from ictus. Measurements at other timepoints also revealed strong correlation between mRS and S100B and moderate correlation between EQ-5D_index_ and S100B.

**Table 2:** EQ-5D outcome at 1-year follow-up

Mobility, *n* (%)	
No problems	49 (74)
Moderate problems	15 (23)
Severe problems	2 (3)
Self-care, *n* (%)	
No problems	53 (80)
Moderate problems	8 (12)
Severe problems	5 (8)
Usual activities, *n* (%)	
No problems	44 (67)
Moderate problems	14 (21)
Severe problems	8 (12)
Pain/discomfort, *n* (%)	
No problems	33 (50)
Moderate problems	27 (41)
Severe problems	6 (9)
Anxiety/depression, *n* (%)	
No problems	35 (53)
Moderate problems	30 (45)
Severe problems	1 (2)
EQ-5D index, mean ± SD	0.58 ± 0.38
EQ-VAS, mean ± SD	72 ± 21.8

*EQ-5D* EuroQoL health-related quality of life measure (3-level version), *EQ-VAS* EuroQol visual analogue scale, *SD* standard deviation

**Table 3:** Spearman correlation between S100B levels and mRS, EQ-5D_index_, EQ-VAS and HH at admission

		Peak	dPeak^a^	Peak d0–1	Peak d0–2	Peak d0–3	Mean d0–10	Mean d0–3	0–12 h	Day 0	Day 1	Day 2
mRS												
	Rho	0.57*	0.62*	0.47*	0.51*	0.50*	0.53*	0.50*	0.57*	0.46*	0.46*	0.40**
	*n*	89	89	79	87	88	89	88	51	65	59	58
EQ-5Dindex												
	Rho	−0.49*	−0.52*	−0.42*	−0.44*	−0.44*	−0.42*	−0.41*	−0.54*	−0.39**	−0.46**	−0.25
	*n*	78	78	70	76	77	76	77	44	57	52	49
EQ-VAS												
	Rho	−0.19	−0.16	−0.12	−0.18	−0.16	−0.12	−0.08	−0.13	−0.05	−0.16	−0.08
	*n*	66	66	58	64	65	66	65	34	45	45	40
HH^b^												
	Rho	0.50*	0.46*	0.57*	0.45*	0.45*	0.53*	0.47*	0.66*	0.53*	0.42**	0.28***
	*n*	78	78	68	76	77	78	77	44	56	51	50

*mRS* modified ranking scale, *EQ-5D, EQ-5D-3L* EuroQoL health-related quality of life measure (3-level version), *EQ-VAS* EuroQoL visual analogue scale, *HH* Hunt and Hess grading scale score. ^a^Dichotomized groups with S100B peak value of ≥ 0.4 μg/l or < 0.4 μg/l. ^b^At admission. *Significant at *p* ≤ 0.001, **significant at *p* ≤ 0.01, ***significant at *p* ≤ 0.05

**Fig. 1:**
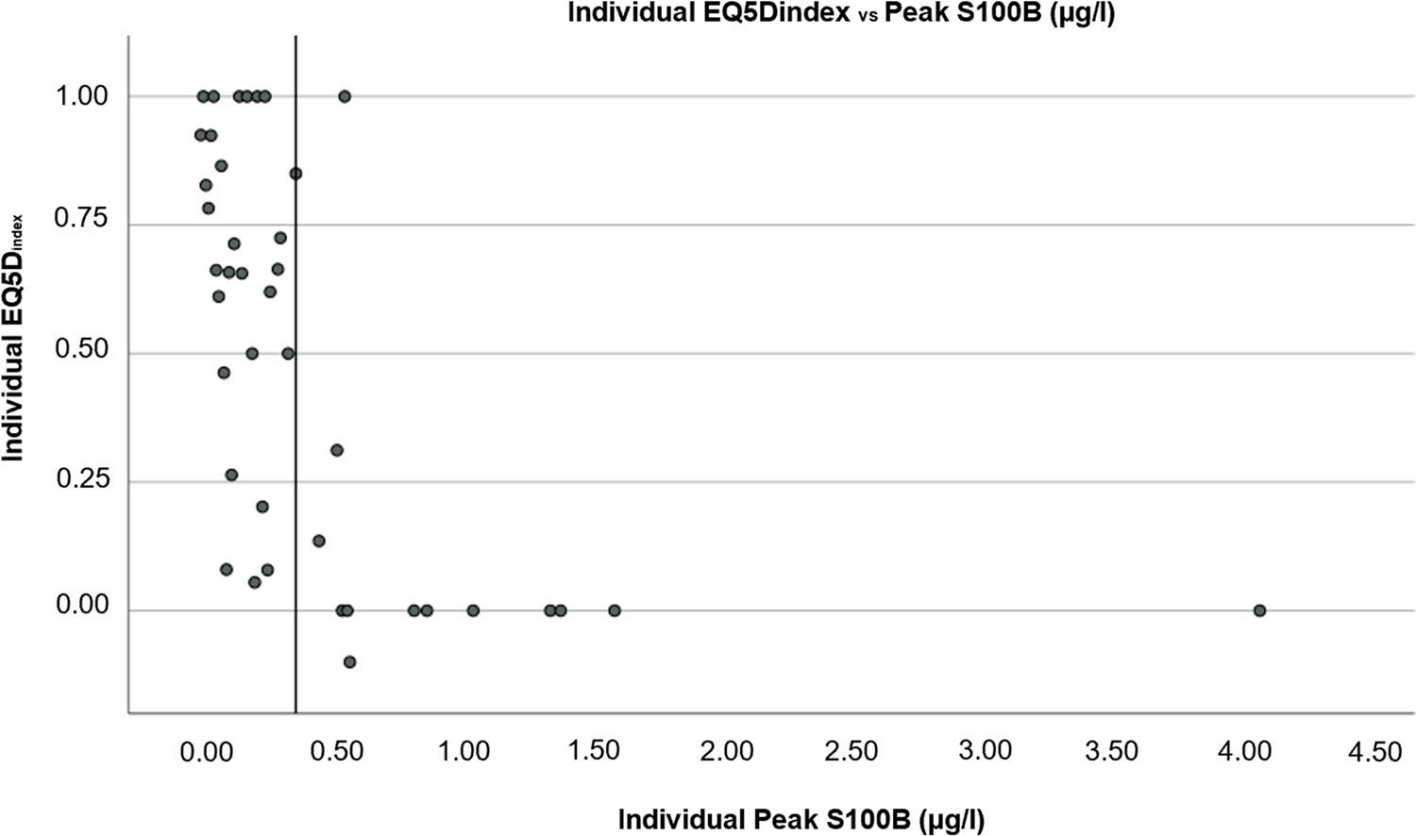
Scatterplot showing individual EQ-5D index score and Peak S100B concentrations for each patient. An x-axis reference line at peak S100B of 0.4 μg/l is shown

Figure 1 illustrates individuals’ EQ-5D_index_ 1 year after bleeding in relation to peak S100B. A reference line illustrates the cutoff for peak S100B concentrations (higher or lower than 0.4 μg/l). Median EQ-5D_index_ at 1-year follow-up was 0.73 (IQR 0.41) for patients with peak S100B values ≤ 0.4 μg/l and 0.00 (IQR 0.07) for patients with peak S100B > 0.4 μg/l (*p* = 0.002).

**Fig. 2:**
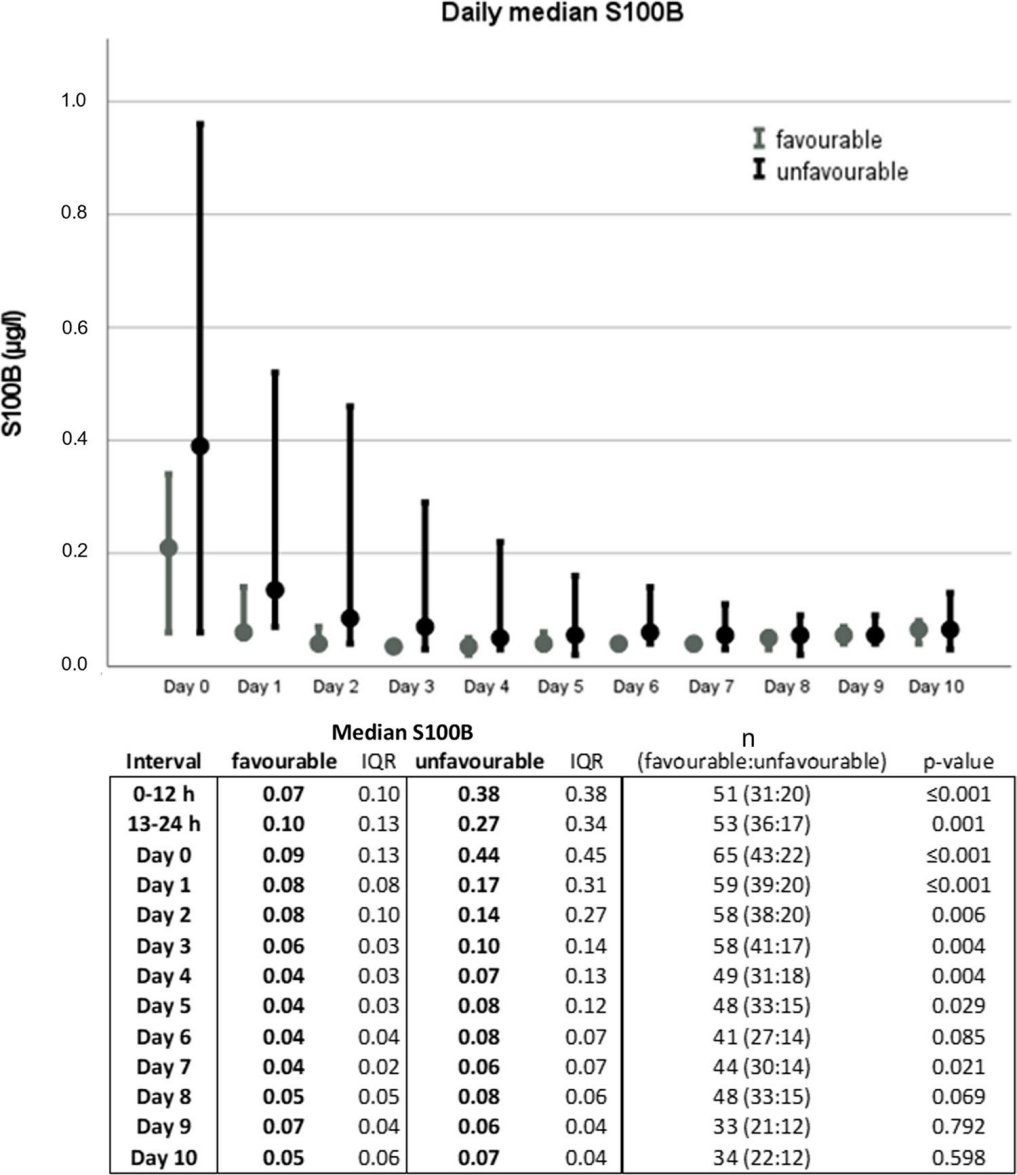
Daily median S100B for dichotomized modified ranking scale (dmRS) groups. Favourable = mRS 0–3 and unfavourable = mRS 4–6. Day 0 represents the first 24 h from ictus. The first 24 h is shown in 12-h intervals in the table, though not represented in the graph. S100B levels shown as μg/l. IQR = interquartile range. Error bars represent 95% confidence interval

Daily median S100B values for the two dmRS groups over time are shown in Fig. 2. There was a significant difference between outcome groups at different timepoints. The highest median S100B value was observed on day 0 in both dmRS groups. Multivariariate logistic regression was used to find clinically relevant predictors for unfavourable outcome defined based on dmRS (Table 4). HH, age, presence of multiple aneurysms, rupture of anterior- versus posterior-circulation aneurysms and peak S100B dichotomized to > 0.4 μg/l or ≤ 0.4 μg/l were considered clinically relevant to include. In this multivariate model, only S100B was a significant predictor (*p* < 0.05). Univariate logistic regression results are also shown in Table 4. In the univariate model, only HH and S100B were significant predictors of outcome. Nagelkerke’s pseudo *R*^2^ revealed a stronger correlation between peak S100B and unfavourable functional outcome as compared with mean values.

**Table 4:** Univariate and multivariate logistic regression analyses for 1 year dichotomized modified ranking scale (mRS) outcome (*ant* anterior cerebral circulation, *post* posterior cerebral circulation)

	Variable	*p*	*n*	Nagelkerke’s pseudo R2
Multivariate			78	0.903
	Hunt and Hess	0.06		
	Age	0.08		
	Multiple aneurysms	0.08		
	Ruptured ant. vs post. circ. aneurysm	0.18		
	S100B > 0.4 μg/l vs ≤ 0.4 μg/l	0.05		
Univariate				
	Hunt and Hess	≤ 0.0001	78	0.556
	Age	0.01	89	0.121
	Multiple aneurysms	0.016	89	0.089
	Ruptured ant. vs post. aneurysm	0.265	89	0.019
	Fisher	0.998	89	0.265
	Hypertension	0.404	89	0.011
	Body mass index	0.473	82	0.010
	Ongoing smoking	0.082	86	0.052
	S100B > 0.4 μg/l vs ≤ 0.4 μg/l	≤ 0.0001	89	0.513
	Peak S100B	≤ 0.0001	89	0.556
	Peak S100B d0–1	≤ 0.0001	79	0.475
	Peak S100B d0–2	≤ 0.0001	87	0.490
	Peak S100B d0–3	≤ 0.0001	88	0.492
	Mean S100B d0–10	≤ 0.0001	89	0.427
	Mean S100B d0–3	≤ 0.0001	88	0.405

Although Fisher grade was not found to correlate with dmRS outcome, peak S100B concentration differed significantly between dichotomized Fisher grade groups (dFG0–3 and dFG4). The median level of peak S100B was 0.09 μg/l (IQR 0.05) in the dFG0-3 and 0.23 μg/l (IQR 0.45) in dFG4 (*p* ≤ 0.001). Within the dFG4 group, there was a significant (*p* ≤ 0.001) difference in median peak S100B between favourable (0.14 μg/l, IQR 0.175) and unfavourable mRS outcomes (0.56 μg/l, IQR 0.745).

Median peak S100B values did not differ significantly between patients given microsurgical (*n* = 49, peak S100B 0.12, IQR 0.15) or endovascular treatment (*n* = 36, peak S100B 0.17, IQR 0.28), *p* = 0.338.

**Fig. 3:**
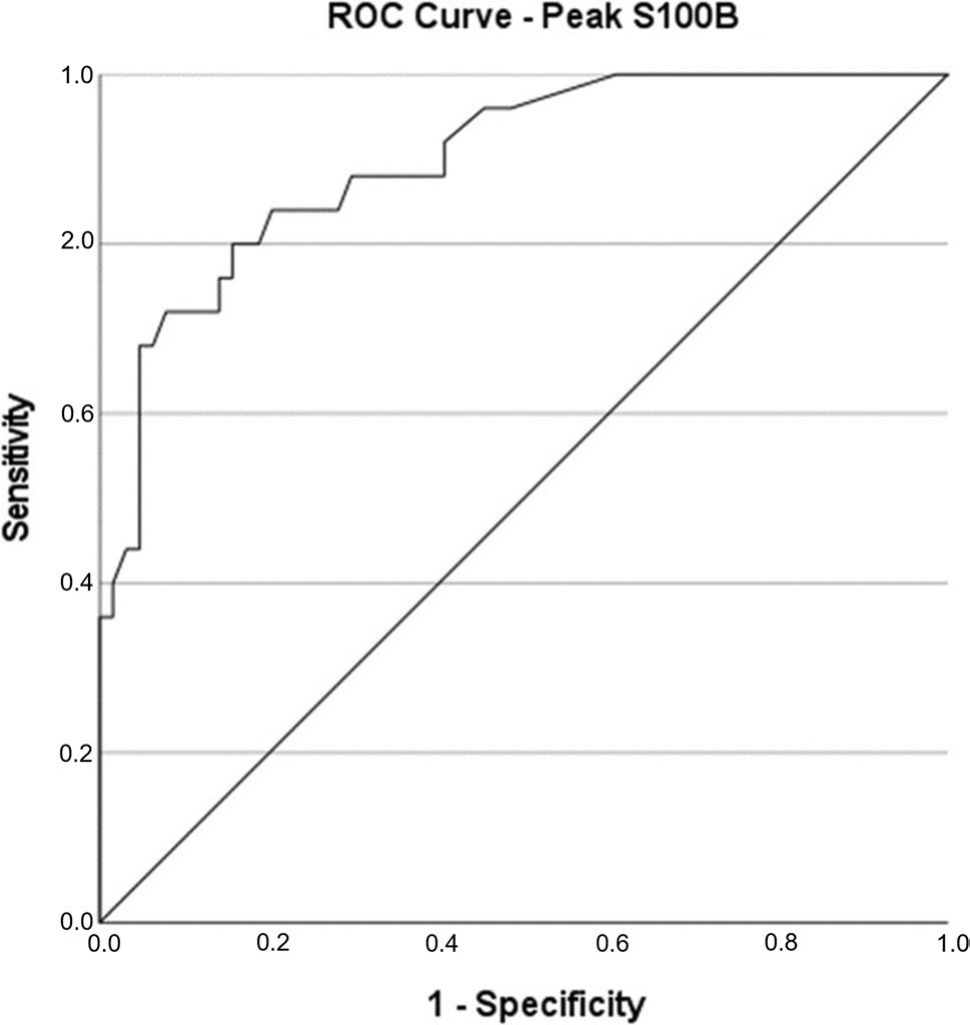
ROC curve showing peak S100B in relation to unfavourable outcome, based on dmRS. AUC 0.898, *p* ≤ 0.0001 (0.828–0.968) (dmRS = dichotomized modified ranking scale, favourable = mRS 0–3 and unfavourable = mRS 4–6. ROC = receiver operating characteristic, AUC = area under the curve)

A ROC curve analysis for peak S100B values (AUC 0.898, 95% confidentiality interval (*CI*) 0.828–0.968, *p* < 0.0001) with a cutoff set at 0.4 μg/l yielded 95.3% specificity and 68% sensitivity for predicting unfavourable outcome based on dmRS (Fig. 3).

Post-hoc analyses were performed to compare data for the patients with sampling every third day (group 1) to those sampled more often due to added clinical sampling (group 2), see Table 5. Further post-hoc analyses were performed comparing the sampling groups where only data from patients with a first S100B sample taken within 24 h of bleeding were included in the analyses, see Table 5. A ROC curve analysis determining unfavourable outcome in correlation to peak S100B was performed including all patients (groups 1 and 2 analysed together) in whom the first S100B sample was taken within 24 h of bleeding (*n* = 65). An AUC of 0.901 (95% *CI*: 0.82–0.981) and a cutoff value of 0.4 μg/l generated 97.7% specificity and 68.2% sensitivity.

**Table 5:** Comparison of time to first S100B sample from bleeding, time to peak S100B value after bleeding and median peak S100B value between group 1 and group 2

		*n*	Time from bleeding to 1st sample (h)	IQR	*p*	Time from bleeding to peak S100B (h)	IQR	*p*	Peak S100B (μg/l)	IQR	*p*-value
A	Group 1	31	42	34		45	63		0.10	0.12	
	Group 2	58	9	5	≤ 0.001	23	29	0.001	0.20	0.39	0.004
	All	89	11	22		33	15		0.16	0.28	
B	Group 1	12	18	4.8		21	34.5		0.12	0.08	
	Group 2	53	8	4.5	≤ 0.001	22	28	0.477	0.19	0.40	0.110
	All	65	9	9		22	22		0.16	0.16	

The two groups are compared: A - for all patients included in the study and B - for patients where the first S100B sample was taken within 24 h of bleeding. All measurements presented as median values. Group 1: S100B samples taken every third day. Group 2: S100B samples taken every third day with addition of routine S100B samples from the clinic

## Discussion

The aim of this study was to evaluate if serum S100B, collected in the acute phase after bleeding, could be used to predict health-related quality of life and functional outcome in patients 1 year after aSAH. Serum levels of S100B after aSAH correlated with unfavourable outcome in both scoring systems. We found that EQ-5D_index_ (but not EQ-VAS) was significantly negatively correlated with serum levels of S100B. The median EQ-5D_index_ at 1-year follow-up differed significantly between patients with a peak S100B level of more or less than 0.4 μg/l.

S100B concentration in serum could be affected by blood brain barrier permeability and glymphatic clearance from cerebrospinal fluid (CSF) to serum [23]. Nevertheless, it can be reliably measured in both serum and CSF [11]. A correlation of S100B in CSF and serum with Fisher grade has been shown, as well as with clinical and functional outcome [1, 8, 11, 13–15, 20, 25, 26]. In our study, Fisher grade alone was not a significant predictor for outcome, but a significant correlation between peak values of S100B and dFG was found. Also, within dFG4, there was a significant difference in S100B values between patients with favourable and unfavourable mRS outcome.

Our study showed an EQ-5D_index_ value of 0.58 ± 0.38 at 1-year follow-up after aSAH. This is in line with results from a larger study, presenting a mean EQ-5D_index_ value of 0.58 ± 0.39 for 755 Swedish aSAH patients [18]. S100B values correlated more strongly with mRS outcome than with EQ-5D_index_ in our results. This might be a result of a power problem, due to a lower number of patients answering the EQ-5D evaluation than with data on mRS. It is also probable that patients with more cognitive deficits are less prone to answer the EQ-5D questionnaire. For EQ-VAS, no deceased patients are included. It is possible that differences in S100B values would not have been sensitive enough to differentiate amongst patients who were doing well enough to respond to the EQ-VAS, even if a larger number of participants had been evaluated. It has been shown by several authors that unfavourable outcome, based on dichotomized Glasgow Outcome Score (GOS), corresponds with significantly higher S100B levels [1, 11, 14, 17, 20, 25]. Our data were well in line with those findings. The mRS is similar to GOS, used for grading functional outcome in patients after aSAH [21]. We chose a dichotomization of mRS that would distinguish between patients in a very poor condition and those who might be in a good clinical condition though somewhat disabled. In many studies, mRS 3 is considered as poor outcome, but there are also studies using the same dichotomization of outcome as we have used [16, 21]. The definition ‘moderate disability, requiring some help, but able to walk without assistance’ led our research group to argue that many patients in this clinical status might have a good life. We therefore chose to include mRS 3 in the ‘favourable outcome’ group. It has been debated if single early S100B samples can be used for prognostication or if a mean value of samples collected over several days needs to be investigated for a realistic view with regard to secondary insults such as vasospasm [25]. Some authors have also claimed that secondary injury to retractors during surgery might release S100B [25]. Our research indicates that several approaches could be used, although we do not consider median values over a longer period to be a feasible clinical tool. Based on our data, there is no obvious advantage from adding later values for prognostication. There was no significant difference in levels of S100B in patients treated with surgery and those given endovascular treatment. Measurements during the first 72 h of ictus seemed to correlate best with functional outcome by dmRS. From day 3 (96 h from ictus), median values for both groups were normalised.

After about one-third of the study period, serum S100B was incorporated as a standard laboratory measure in our clinical setting. This resulted in a substantial number of extra samples for about two-thirds of the study group. Post-hoc analyses showed that the first sample was taken significantly earlier and the median peak S100B was significantly higher in the latter group. When sorting out patients with the first S100B sample taken within 24 h of bleeding, the difference in median peak S100B concentration did not remain and the median time from bleeding to measured peak S100B did not differ between the groups. A post-hoc ROC curve analysis and regression analysis for the group with the first S100B sample taken within 24 h of bleeding showed an even stronger correlation between median peak S100B and functional outcome by dmRS as compared with the original patient cohort.

The best ROC curve was generated using peak S100B. A cutoff level of 0.4 μg/l yielded 95.3% specificity and 68% sensitivity for predicting unfavourable outcome. Our results may be compared to those amongst patients evaluated at discharge from intensive care [11] reporting a cutoff value of 0.23 μg/l for peak S100B with an AUC of 0.837 and 73% specificity and 85% sensitivity for unfavourable outcome (GOS 1–3). Another study suggests a S100B cutoff level of 0.4 μg/l, for mean daily values on the first 8 days after aSAH [25]. In that study, the AUC was 0.80, specificity 87% and sensitivity 50%. A third study reported mean daily S100B values on the first 15 days to be the most reliable value for predicting functional outcome. That study showed an AUC of 0.98, 95% *CI* (0.87–0.99), 91% sensitivity and 90% specificity with a cutoff of 0.23 μg/l [20]. For comparison, we performed a post-hoc ROC analysis using the mean S100B on days 0–10 and a cutoff value of 0.215 μg/l. This resulted in an AUC of 0.863 (*p* ≤ 0.001), 95.3% specificity and 52% sensitivity for predicting unfavourable outcome based on dmRS.

In our study, peak values of S100B corresponded to a more reliable ROC curve as compared with mean values for days 0–10. This might reflect fewer patients presenting with late complications such as vasospasm in our dataset. The post-hoc analysis performed to compare patients with samples taken every third day and those in whom samples were taken more frequently underlined that early sampling is important to catch the peak S100B value.

The multivariate logistic regression analysis was applied to evaluate the relationship between dichotomized S100B and unfavourable outcome after adjusting for other clinically relevant variables. Body mass index was considered, but discarded due to difficulties achieving correct measures of both height and weight in the acute situation. Hypertension was also discarded due to large differences in severity between individuals, which cannot easily be assessed in the acute setting. Smoking habits would also be of interest but cannot be assumed to be available. Lastly, the Fisher grade was discussed, but discarded. Insensitivity of the instrument and differences in interpretation due to atrophy of the brain and its intended use to predict occurrence and severity of vasospasm, rather than functional outcome, led to this decision. In the multivariate model, only dichotomized S100B was found to be significant. The univariate analysis found HH and age, as well as S100B values, to be significant predictors of unfavourable outcome [9].

Our study had some limitations and advantages. A built-in drawback of using self-assessment tests for correlation with a biomarker is that patients with impaired communication abilities will have more trouble filling out forms. S100B may not have sufficient sensitivity for identification of differences between the patients healthy enough to answer a questionnaire and/or the questions may not pinpoint all the issues that are relevant. Due to prospective collection of data (2014–2018), changes in clinical practice have had an impact on the study. Part of the collection of S100B samples was not blinded to the clinicians and a certain bias might be suspected. A timing and frequency difference in sampling over time has occurred. This might have affected the peak value of S100B, which was significantly lower in the group of patients with sampling done only every third day.

Suggestions for future studies include collecting a larger, well-defined cohort of aSAH patients with a strict protocol for sampling of S100B. A multicentre study would be preferable, to minimise risk of bias due to clinical decisions. We suggest that peak S100B within the first 72 h of bleeding may be a prognostic factor for functional outcome. The use of EQ-5D as a tool to investigate the quality of life in patients with aSAH needs to be explored further.

## Conclusions

We found a clear correlation between peak S100B levels in serum after aSAH and outcome. An S100B value over 0.4 μg/l seemed to have a strong correlation with unfavourable functional outcome.

The results could be used to help clinicians estimate clinical outcome. A future multicentre study would be important to confirm or reject our results and to establish a cutoff value of S100B in patients with aSAH. This would be used for prognostication in clinical practice.
